# A Soft Computing-Based Analysis of Cutting Rate and Recast Layer Thickness for AZ31 Alloy on WEDM Using RSM-MOPSO

**DOI:** 10.3390/ma15020635

**Published:** 2022-01-15

**Authors:** Kapil K. Goyal, Neeraj Sharma, Rahul Dev Gupta, Gurpreet Singh, Deepika Rani, Harish Kumar Banga, Raman Kumar, Danil Yurievich Pimenov, Khaled Giasin

**Affiliations:** 1 Department of Industrial & Production Engineering, Dr. B. R. Ambedkar National Institute of Technology, Jalandhar 144805, India; kapilacad@gmail.com; 2 Department of Mechanical Engineering, Maharishi Markandeshwar (Deemed to be University), Mullana 133207, India; neerajsharma@mmumullana.org (N.S.); rdgupta@mmumullana.org (R.D.G.); 3 Department of Automobile Engineering, Amity Institute of Technology, Amity University, Noida 201313, India; gurpreet.er85@gmail.com; 4 Department of Mathematics, Dr. B. R. Ambedkar National Institute of Technology, Jalandhar 144805, India; ranid@nitj.ac.in; 5 Fashion and Lifestyle Accessory Design Department, National Institute of Fashion Technology, Mumbai 410210, India; drhkbanga@gmail.com; 6 Department of Mechanical and Production Engineering, Guru Nanak Dev Engineering College, Ludhiana 141006, India; 7 Department of Automated Mechanical Engineering, South Ural State University, Lenin Prosp.76, 454080 Chelyabinsk, Russia; danil_u@rambler.ru; 8 School of Mechanical and Design Engineering, University of Portsmouth, Portsmouth PO1 3DJ, UK

**Keywords:** AZ31 alloy, hybrid approach, Multi-Objective Particle Swarm Optimization (MOPSO), recast layer, response surface methodology (RSM), wire-cut electric discharge machining (WEDM)

## Abstract

In the present research, the AZ31 alloy is machined by wire-cut electric discharge machining (WEDM). The experiments were designed according to the Box-Behnken design (BBD) of response surface methodology (RSM). The input process variables, namely servo feed (SF), pulse on-time (Ton), servo voltage (SV), and pulse off-time (Toff), were planned by BBD, and experiments were performed to investigate the cutting rate (CR) and recast layer thickness (RCL). The analysis of variance (ANOVA) was performed to determine the influence of machining variables on response characteristics. The empirical models developed for CR and RCL were solved using Multi-Objective Particle Swarm Optimization (MOPSO). Pareto optimal front is used for the collective optimization of CR and RCL. The optimal solution suggested by the hybrid approach of RSM-MOPSO is further verified using a confirmation test on the random setting indicated by the hybrid algorithm. It is found that the minimum RCL (6.34 µm) is obtained at SF: 1700; SV: 51 V; Toff: 10.5 µs; and Ton: 0.5 µs. However, maximum CR (3.18 m/min) is predicted at SF: 1900; SV: 40 V; Toff: 7 µs; and Ton: 0.9 µs. The error percentage of ±5.3% between the experimental results and predicted solutions confirms the suitability of the proposed hybrid approach for WEDM of AZ31.

## 1. Introduction

WEDM is a non-traditional machining technique that can effectively generate intricate shapes from conductive materials [1]. WEDM is a thermal-erosion process [2], where localized heating is utilized to remove the material, which is caused due to plasma channel. Plasma is produced between the workpiece and electrode [3]. Only a fraction of the heat from the total produced is transferred to the workpiece to melt the material and evaporate it, which is further fleshed out while utilizing de-ionized fluid flowing. After that, a carter is left on the machined surface [4]. In WEDM, a discharge cycle with very short Toff and Ton causes a quick quenching and heating effect on the machined surface [5]. This process became suitable for the processing of difficult to machine and conductive material. The superalloy Monel K-500 is processed by Agarwal et al. [6] on WEDM varying the Toff, Ton, SV, and wire feed. It was found from their research that Toff, Ton, and SV have a significant influence on cutting speed and SR, while wire feed has a negligible effect on the responses. Another research to process nickel-based superalloy (Inconel 800) was done by Sen et al. [7] using WEDM. The process parameters are optimized using an Analytical Hierarchy Process integrated with Additive Ratio Assessment. It has been found from their research that the optimized setting suggested by the proposed method shows an excellent result reproducibility. One research has been conducted on the hybrid composite (LM25/fly ash/B4C) by optimizing the process parameters of WEDM. It was observed that grass hopper optimization outperformed other optimization techniques such as particle swarm optimization and moth-flame optimization [8]. WEDM is also successfully used for the machining of biomedical alloys [9]. Another category for the processing of biomedical alloys by WEDM includes biodegradable material. The potential materials for the biodegradable implants are magnesium and its alloys because of their mechanical properties, which are biocompatible [10]. Mg and its alloys have good mechanical characteristics (100–250 MPa) that are suitable for an implant, Young’s modulus (41–45 GPa) equivalent to that of human bone, and low density (1.74–2.0 g/cm^3^) [11]. In the last few years, different biodegradable implants have been developed using Mg alloys, which showed great resistance toward corrosion and mechanical strength [12,13,14,15,16,17]. However, in the physiological environment, the high corrosion rate of such alloys is the chief obstacle to developing degradable implants [15,16]. To decrease the corrosion rate, surface coatings [13,14] can be considered to reduce the chances of implant failure [12]. Xu et al. [18] studied the effect of WEDM on the corrosion rate and surface morphology of AZ91D. Wire EDMed resulted in micro-pits, high surface roughness, and micro-cracks, decreasing as the power tube number increases. The Energy-Dispersive X-ray (EDX) analysis demonstrated that AZ91D alloy expressed excellent adhesion strength and better corrosion resistance when the carbonaceous layer formed on it. Sun et al. [19] developed a protective layer by micro-EDM, which enhances the corrosion resistance.

Along with developing a protective layer, the optimization of WEDM reduces the micro-pits and micro-cracks [20]. Yoo et al. [21] established the relation between the corrosion resistance and mean surface roughness (Ra) of AZ91 Mg alloy. It has been found that the value of Ra increases with the current leakage. Walter et al. [22] machined AZ91 alloy and analyzed the effect of Ra on polarization resistance. At a high value of Ra, the polarization resistance decreases by 30%. Song and Xu [23] investigated that polishing of grinded AZ31 alloy ameliorates corrosion resistance. Yue et al. [24] worked on four processes: WEDM, single-point diamond grinding, polishing operation, and grinding. The corrosion potential and surface quality of the Mg composite are investigated for all processes. Another research study carried by Siddiqui and Ramkumar [25] worked on AZ31 alloy by micro-WEDM to investigate kerf width and material removal rate. They found that voltage and capacitance play a major role rather than wire tension and wire feed for the processing of biomedical alloy. In another work, the researcher evaluated the corrosion rate [26] after the WEDM of AZ31 alloy and observed that the surface processed at high discharge energy parameters developed micro-cracks and a recast layer. This surface was very prone to corrosion, which decreased the corrosion resistance of the material.

In the last few years, several researchers made attempts to optimize the process parameters of WEDM considering multiple performance characteristics using gray relational analysis [27], utility concept [28], desirability [29], and artificial intelligence (AI) [30] techniques. Out of those mentioned above, AI techniques are preferred due to their quick response with a near-optimal solution. The statistical approaches were used for the planning of experiments and analysis of results by simple mathematical processes. However, the results obtained by these methods require the proper selection of weights and other constants. The AI techniques used for the optimization of process parameters need a large database to solve the problems. Once a significant number of experiments were performed for some response variables, the prediction of the solution became easy and fast by AI intelligence techniques. Here, in the present work, the experiments were planned by statistical approach, and solutions were predicted by MOPSO. The results obtained by MOPSO are quick compared to genetic algorithms and non-dominated genetic algorithm.

Only a little amount of research has been done on the WEDM machining of magnesium alloys. This WEDM can be considered an effective route to develop an Mg-based implant. To ensure that WEDM is the best technique for establishing implants (Mg alloy), researchers are working on the WEDM of magnesium alloys for corrosion behavior and other characteristics. Optimal settings of WEDM parameters are needed to be ensured for the minimum corrosion rate and better surface properties. Therefore, the effect of WEDM on the response variables of AZ21 alloy is explored. In the literature review regarding the machining of Mg alloys and their parametric optimization, there has been more significant research in the open literature on the optimization of machining process variables of AZ31 using a statistical approach. However, limited research was published on the hybrid optimization (combination of statistical and AI approaches) of AZ31 alloys using RSM-MOPSO for WEDM: especially, a combination of CR (larger than the better) and RCL (smaller the better). Therefore, in the present work, AZ31 alloy is machined by WEDM, considering CR and RCL as response characteristics.

The rest of the paper is organized after reviewing the significant literature in Section 1. Section 2 consists of Materials and Methods, including the research steps followed, experimental setup, materials utilized, and experimental array. Section 3 describes the methodologies used, such as RSM, MOO, PSO, and the implementation procedure of the proposed MOPSO. Section 4 consists of Results and Discussion, including analysis of CR and RCL and confirmation of experiments to verify the attained optimal results. Finally, Section 5 depicts the morphological analysis of machined surfaces, which is followed by conclusions based on the achieved results in Section 6.

## 2. Materials and Methods

In the present research, AZ31 Mg alloy of the biomedical grade was used for the experimentation. The workpiece has the dimensions of 200 mm × 200 mm × 6 mm, and the specimen size extracted from this plate is 10 mm × 10 mm × 6 mm. The physical and mechanical characteristics of the work material used in the present work are given in Table 1 [31]. In addition, Table 2 depicts the chemical composition of the material used in the current research [31].

The current research progresses as per the following steps:

I. The SF, SV, Ton, and Toff are input machining variables planned per the RSM-based BBD to develop one experimental array.
II. The response characteristics (i.e., CR and RCL) are measured corresponding to the experimental array.
III. The analysis is to be carried out for each response, and the mathematical models are to be developed for the response characteristics.
IV. Hybrid optimization is to be processed by the MOPSO on the mathematical models developed by RSM.
V. Validation experiments are to be performed on the random predicted solution suggested by the optimal Pareto front.
VI. The surface morphology of the machined part (machined at the optimal setting suggested by the hybrid approach) is also studied for its features.

### 2.1. Experimental Setup

**Figure 1:**
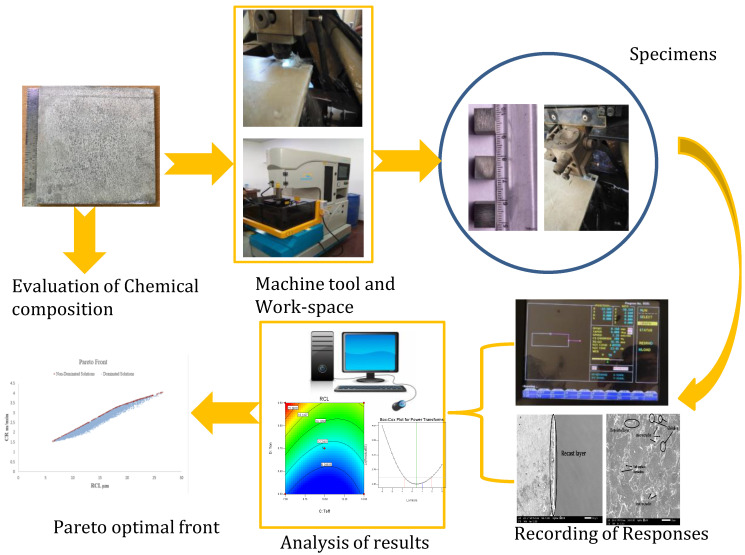
Process of research adopted in present work.

The Electronica (Pune, India) make Ecocut (model: Elpuls15) WEDM was used to conduct the current research on AZ31 alloy. The machine tool has several input process variables, and out of all the available variables, SF, SV, Ton, and Toff were selected after the preliminary study. All the experiments were conducted in the presence of dielectric. In the present research, de-ionized water was utilized as a dielectric to eliminate the micro-chips of material from the spark gap. The brass wire (diameter 0.25 mm) is being used as an electrode. Some weight terms minimized the deflections in the traveling wire as wire tension. Some parameters during the experiments were kept fixed: wire tension: 7 N, flushing pressure: 6 kg/cm^2^, conductivity: 20 mho, temperature: 23 °C; wire feed: 9 m/min; peak current: 10 machine units. The complete research process in the current work is presented in Figure 1.

The cutting rate (CR) and recast layer (RCL) were measured during the machining of AZ31 alloy. The cutting rate was observed (from the display screen of the WEDM) when the wire travels some distance (5 mm) during machining so that machining becomes stable and the machining rate is at its peak. The cutting rate becomes low; consequently, the CR is not recorded near the corners. The RCL value was measured using Jeol (Model: JSM IT500, Japan) make scanning electron microscope. Before the measurement of RCL, the surface of the specimen is prepared with abrasive paper and diamond paste. The abrasive paper of different grades is used for polishing (SETEST, model: SE 1813, India), starting from 1600 mesh to 2400 mesh size. Finally, the diamond paste of 1 μm is used for polishing. The etchant was applied on the surface to observe the microstructure.

### 2.2. Experimental Array

The experimental array is the combination of input process variables according to which the experiments were performed. This experimental array depends upon the various process variables and their setting. In the current research, four process variables, namely SF, SV, Toff, and Ton, are used for experimental purposes. The range of the input machining variables is given in Table 3. The process variables, their ranges, and levels are selected after preliminary experiments.

A Box–Behnken-based experimental array is used in the present research with an identified search space (α = 1). This is considered due to the fact of wire rupture during WEDM. If the input process parameters are extrapolated outside the search space (α > 1), WEDM loses its quality and productivity. Therefore, a preliminary study was initially conducted to keep the input process variables within the operating range. After envisaging the operating range, the designing of experiments be carried out, as depicted in Table 4. All the experiments were performed as per the run order rather than the standard order. This is due to the principle of randomness and checks the machine tool results reproducibility. A total of 29 experiments were performed, and each experiment was replicated twice to maintain the statistical accuracy of the results.

## 3. Methodologies

### 3.1. Response Surface Methodology (RSM)

Box and Wilson developed RSM in 1951 to optimize responses evaluated after the experiments [32]. It is a statistical technique in which experiments are primarily planned; then, according to the setting, experiments were performed and then analyzed to improve the services and processes [33,34]. The current work shows the response characteristics, namely CR and RCL, as functions of process variables in Equations (1) and (2).

(1) $$CR=\emptyset \;\left(A_{1},A_{2},A_{3},A_{4}\right)$$

(2) $$RCL=\varphi \left(A_{1},A_{2},A_{3},A_{4}\right)$$

Equations (1) and (2) in the first of the higher-order model can explain the nature of response characteristics. The drawback in the first order is that the lack of fit exists because of interaction terms and surface curvature. However, in the second-order model, this drawback can be eliminated with a significant improvement in optimization of the process. Finally, after neglecting the higher terms, the second-order model is represented in Equation (3).

(3) $$Response=\in_{0}+\overset{m}{\underset{k=1}{\sum }}\in_{k}A_{k}+\overset{m}{\underset{k=1}{\sum }}\in_{kk}A_{k}^{2}+\overset{m}{\underset{n<k=2}{\sum }}\in_{kn}A_{k}A_{n}$$

where ϵ_0_, ϵ*_k_*, ϵ*_kk_*, and ϵ*_kn_* are regression coefficients;

*m*: total number of process variables.

*A_k_*: *k*th Process variables.

### 3.2. Implementing Multi-Objective Particle Swarm Optimization (MOPSO)

#### 3.2.1. Multiple Objective Optimization (MOO)

Most real-world problems generally include the concurrent optimization of many responses, which are usually conflicting in nature and non-commensurable. No single alternative can be better than all other alternatives in the MOO problem, considering all the objectives [35,36,37]. A general multiple objective optimization problem consists of two or more objective functions and some inequality and equality restraints. The mathematical model of a widespread MOO problem can be represented as follows [38]:

(4) $$\begin{array}{c} {\mathrm{Max}/\mathrm{Min}\;f}_{i}\left(x_{1},x_{2},\ldots ,x_{m}\right)i=1,2,\ldots ,n\\ \mathrm{Subject}\;\mathrm{to}\;\\ g_{j}\left(x\right)\leq 0j=1,2,\ldots ,J\\ h_{k}\left(x\right)=0k=1,2,\ldots ,K \end{array}$$

where x represents the decision variable vector of length m, the problem deals with n number of objectives, J represents the number of inequalities, and K represents the equality restraints. The solution to the MOO problem occurs in terms of different trade-offs termed a Pareto front [39]. For assigning fitness in such a case, the concept of non-domination was introduced by Vilfredo Pareto in 1896 [40].

The following two conditions are mandatory for establishing the fact that a solution X1 leads to solution X2:

1. In all the objectives, solution X1 should not be worse than X2.
2. In at least one of the objectives, the solution X1should be better than X2.

All the non-dominated solutions (NDS) together generate a Pareto front, i.e., the set of alternatives representing the trade-off values of various competing objective functions. Achieving a uniformly distributed true Pareto front is the primary goal of an MOO algorithm. Thus, the NDS that seem equally important can further be assigned a value of importance, i.e., the quality index to maximize the distance from NDS available in the immediate neighborhood along all the objectives to ascertain a uniformly distributed Pareto front.

In various classical optimization techniques, the multiple goals are combined into a composite objective function assigning weights to different competing objectives suggested by the experts. However, such a methodology results in subjectiveness in the decision-making process. Moreover, the management may also be deprived of the complete set of alternatives that might be important in final decision making considering various probable scenarios. In the traditional mathematical programming approaches such as ε-constraint and weighting methods, MOO problems are converted to a single-objective problem. As a result, only one solution can be achieved per optimization effort.

In contrast, the evolutionary algorithms have many benefits over the classical techniques (optimization) [41], which use a group of solutions in every run and result in a set of near-optimal solutions after each run. Such heuristics are based on random initialization and stochastic search to locate the global optima. Therefore, population-based stochastic search techniques are a better choice to solve MOO problems in which numerous NDS increase drastically with the increase in several objectives.

#### 3.2.2. Particle Swarm Optimization (PSO)

Kennedy and Eberhart [42] proposed the PSO algorithm. In PSO, the potential solution is a school of fishes or flock of birds termed a swarm of particles. Each particle has two parameters: velocity and position. Instead of evolution in genetic algorithms, the identical particles keep flying in the search space throughout the iterations by changing their position. Each particle settles its position during the flight as per its own, and its neighboring particles direct the search in the promising region. The best experience of the i^th^ particle achieved so far is represented by $Pbest_{i}$: the global best position attained among all the particles described by Gbest.

Suppose an optimization problem consists of D decision variables formally known as D dimensions of the search space and M number of objectives formally known as fitness functions. Let x represent the particle coordinates, i.e., position, and v represent the flight speed, i.e., velocity in the space (search). The position of the i^th^ particle in the swarm can be presented as a D dimensional vector, i.e.,

$$X_{i}=\left(x_{i}^{1},x_{i}^{2},\ldots ,x_{i}^{d},\ldots ,x_{i}^{D}\right).$$

It embeds the relevant information regarding the D decision variables. Similarly, the velocity of an i^th^ particle can be represented as

$$V_{i}=\left(v_{i}^{1},v_{i}^{2},\ldots ,v_{i}^{d},\ldots ,v_{i}^{D}\right).$$

The position of each particle dictates the fitness value that provides a signal of its execution in the objective space. The following two equations are used to update the position and velocity of each particle during the iterations

(5) $$v_{i,\left(t+1\right)}^{d}=\omega_{\left(t\right)}*v_{i,\left(t\right)}^{d}+c_{1}*rand_{1}\left[Pbest_{i,\left(t\right)}^{d}-x_{i,\left(t\right)}^{d}]+c_{2}*rand_{2}[Gbest_{\left(t\right)}^{d}-x_{i,\left(t\right)}^{d}\right]$$

(6) $$x_{i,\left(t+1\right)}^{d}=x_{i,\left(t\right)}^{d}+v_{i,\left(t+1\right)}^{d}$$

where $x_{i,\left(t\right)}^{d}$ and $x_{i,\left(t\right)}^{d}$ are the position and velocity of the i^th^ particle for the d^th^ dimension in t^th^ iteration. $c_{1}$ and $c_{2}$ are coefficients (acceleration) and the $rand_{1}$ and $rand_{2}$ are the uniform random numbers in the range (0–1). The $\omega_{\left(t\right)}$ represents the inertia weight that sets up a balance between exploitation and exploration. The linearly decreasing value of the inertia weight factor gives better convergence properties.

$$w=w_{\max}-\frac{\left(w_{\max}-w_{\min}\right)t}{t_{\max}}$$

where $w_{max}$ and $w_{min}$ represent a randomly chosen initial and the final value of the inertia weight factor. $t_{max}$ and t denote the maximum number of iterations and the current iteration. Consequently, the inertia weight factor continuously decreases from maximum value to the minimum to initially focus on exploration and slowly move toward exploitation for better convergence. However, the velocity and position updates through Equations (5) and (6) are liable to cause particles to cross the boundaries of the feasible regions. For handling this problem, the location is truncated at the extreme of the boundary, and velocity is redefined to move the particle away from the boundary in upcoming iterations. Various modified versions of MOPSO, along with the selection of parameters and the potential applications, have been discussed by Eberhart and Shi [43] and Shi and Eberhart [44,45].

#### 3.2.3. Multi-Objective Particle Swarm Optimization (MOPSO)

Coello-Coello and Lechuga [46] are among the few pioneers who successfully extended the concept of PSO to attempt MOO problems, and these methods are known as MOPSO. In the latter study, they also compared MOPSO with micro-genetic algorithm [47], Pareto archive evolutionary strategy [48], and non-dominated sorting genetic algorithm-II (NSGA-II) [49] for various test functions. They concluded the strength of MOPSO in covering the full Pareto front [50]. The extension of PSO to MOPSO poses a challenge to allocate the guides to the individual particles; as in a MOO scenario, there is no single optimal solution. Instead, a set of optimal solutions exist, i.e., multiple NDS are preserved after each iteration. In most MOPSO approaches, a set of NDS is maintained in the external repository or archive. The guide is allocated to every individual in the swarm based on various algorithms.

The size of the external repository may be kept at a fixed size or unlimited, which also has a major effect on the outcome of the MOPSO algorithm.

In the present work, the external repository of fixed size is considered to maintain the NDS. After each iteration, the current NDS are stored in the repository. To keep the repository domination free, the dominated solutions must be deleted from the repository.

**Figure 2:**
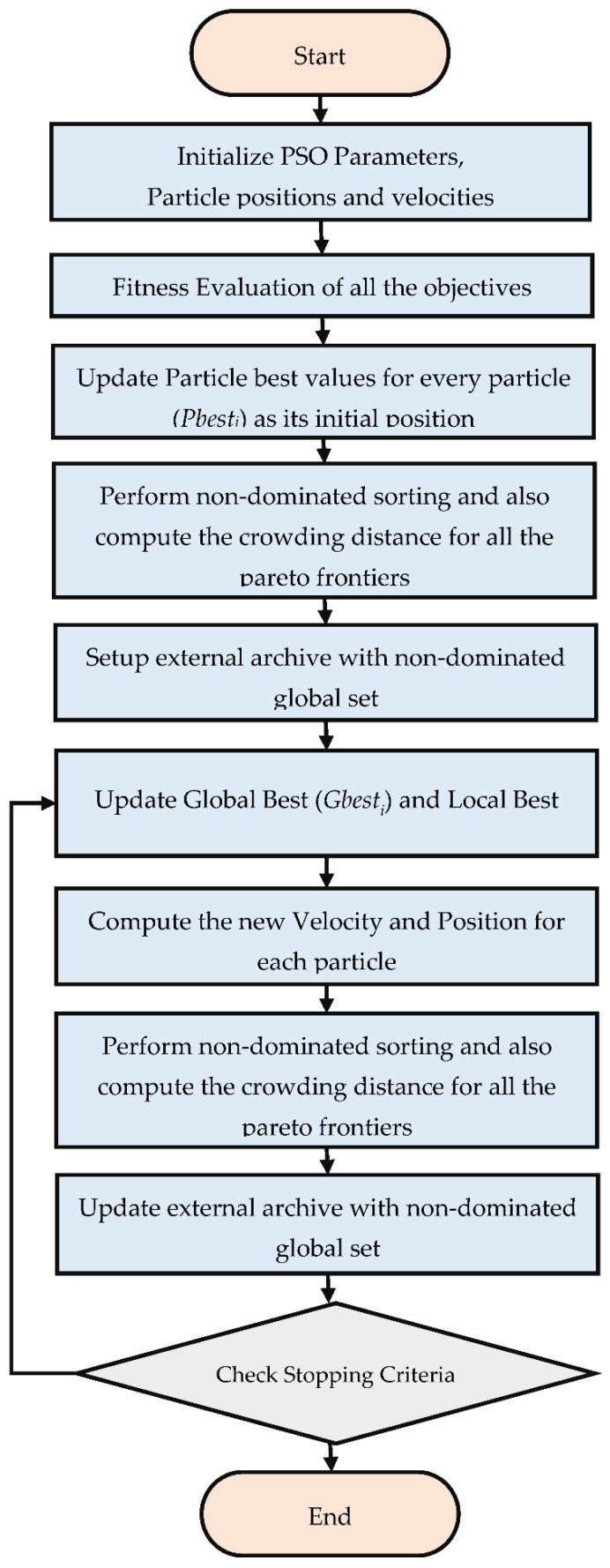
Flow diagram presenting MOPSO algorithm.

To select guides and handle the problem of a limited-size repository, the NDS is further graded to assign higher significance to the solutions located in the less populated areas of the objective space. The process is known as designating the crowding distance to the Pareto frontiers, ensuring the uniform distribution of the Pareto front. Furthermore, the global guide, i.e., Gbest, is assigned to each particle in the swarm through roulette wheel selection based on the crowding distance of Pareto frontiers. Each particle’s local best position is also maintained by preserving the best position of the particle achieved so far; i.e., the Pbest of each particle is also updated after each iteration. If the particle’s current position dominates the previous position, the current position is stored as the new Pbest. When none of the particles dominates, the Pbest is selected randomly. After selecting guides, each particle’s velocity and position is updated according to Equations (6) and (5). The MOPSO algorithm [51,52] implemented in this study is briefly outlined in Figure 2.

## 4. Results and Discussion

The results corresponding to the input process variable setting during the machining of AZ31 alloy are given in Table 4. The attribute of response characteristics is decided according to the quality control and productivity of manufacturing.

### 4.1. Analysis of Cutting Rate (CR)

The summary of analysis (Table 5) shows that the Toff and Ton exhibit *p*-values less than 0.05, due to which these are categorized as influential process variables for CR. On the other hand, the *p*-values in SF and SV are greater than 0.05. Still, these parameters are assumed for the analysis purpose of CR. This is done to maintain the model’s hierarchy, according to which the parameter itself is not significant. Still, its interaction term with other parameters is significant, or its quadratic term is significant. In the present work, the quadratic term of SF and SV shows a *p*-value less than 0.05. The higher the SS value and F-value corresponding to a parameter, the more its percentage impact for the analysis of a particular response will be. Ton shows the highest SS value and F-value [53]. Therefore, the impact of Ton is maximum in the analysis of CR.

**Figure 3:**
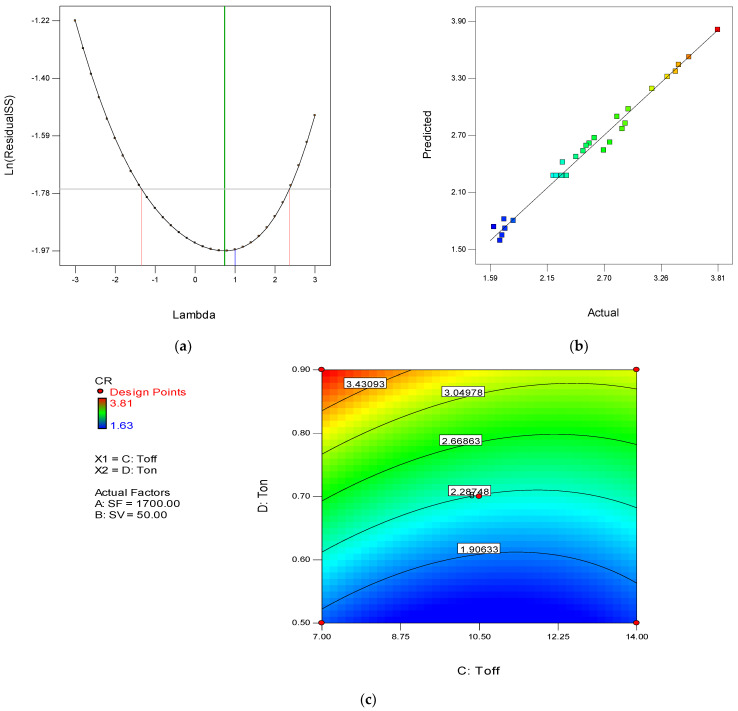
(**a**) Box–Cox transformation for CR. (**b**) Predicted versus actual plot for CR. (**c**) Contour plot for Toff and Ton in case of CR.

The Box–Cox transformation recommends the value of lambda (λ) according to the analysis. With the help of Box–Cox change, an appropriate value for λ is applied to the response data. From Figure 3a, it is clear that the recommended value of λ is 1. Thus, in the power transformation during the analysis, ‘1’ is used instead of any other value. Figure 3b represents the predicted versus actual plot of residuals. It is evident from the plot that all values fall on the straight line, which is a sign of a suitable ANOVA. The contour plot depicted in Figure 3c is the interaction plot of Toff and Ton. The red color in the plot shows a CR value equal to 2.81 m/min. However, the blue color indicates a 0.63 m/min CR value. On the contour line, the CR value is also defined. It is clear from the plot that the maximum CR is obtained when Toff is minimum and Ton is maximum (as red color found on the top left corner of the plot) [54].

Similarly, minimum Ton and maximum Toff show blue color. Thus, the CR is nearby 0.63 m/min. The mathematical model developed after the analysis for CR is given in Equation (7).

CR = +14.67050 − 7.92625E − 003 × SF − 0.23775 × SV − 0.34581 × T_off_ + 2.39875 × T_on_ − 0.19286×T_off_ ×T_on_ + 2.37292E − 006×SF^2^ + 2.33667E – 003 × SV^2^ + 0.020503 × T_off_^2^ + 2.81042 × T_on_^2^ (7)

**Figure 4:**
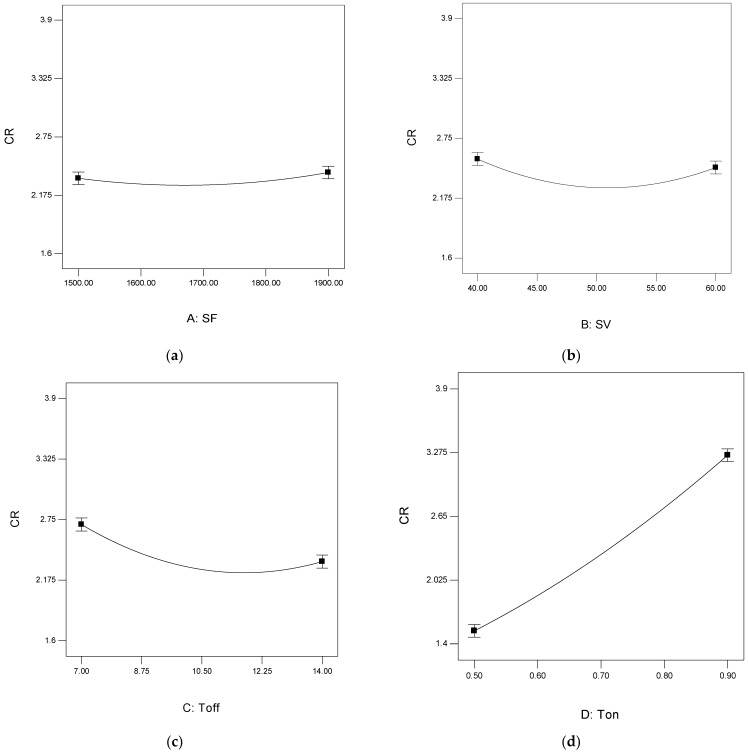
Graphs for CR analysis. (**a**) Variation of CR with SF; (**b**) Variation of CR with SV; (**c**) Variation of CR with Toff; (**d**) Variation of CR with Ton.

Figure 4 depicts the variation of CR with respect to the process parameters. Figure 4a shows that with the increase in SF, a slight rise in CR (from 2.34 to 2.39 mm/min) is observed. This may be because the new wire comes in contact with the work material with increased SF value. With the increase in SF, the new cutting characteristic wire removes the extra material, which increases the CR. Figure 4b shows the variation of CR concerning the SV. It was found that with the increase in SV, the CR decreased from 2.55 to 2.46 mm/min. Similar trends were observed while machining the Ti-6Al-4V by Gupta et al. [55]. At a low value of SV, the gap between tool and workpiece is also low. At this point, when the current is supplied, excessive material is removed.

Similarly, at a high value of SV, the same amount of current removes less material. Figure 4c presents the plot between Toff and CR. It was observed that with the increase in Toff value, the CR decreases from 2.7 to 2.35 mm/min. However, the research findings are in line with the findings of Sharma et al. [56] while machining Ti-6Al-4V. The probable reason is that with the increase in Toff value, the current off-time in the circuit increases, which decreases the discharge energy in the circuit and hence decreases the CR.

Similarly, with the rise in Ton value, the circuit’s current on-time increases, increasing the discharge energy and CR, as shown in Figure 4d. Here, the CR was increased from 1.52 to 3.25 mm/min. A similar trend of the variation of CR concerning Ton was observed by Sharma et al. [56].

### 4.2. Analysis of Recast Layer Thickness (RCL)

The summary of RCL (Table 6) shows that Toff, Ton, the interaction of Toff and Ton, and the quadratic terms of SF, SV, Toff, and Ton significantly affect the model of RCL. The developed model of RCL is significant; however, the lack of fit is not significant. Ton shows the maximum influence on RCL, which is followed by Toff, SV, and SF [53].

**Figure 5:**
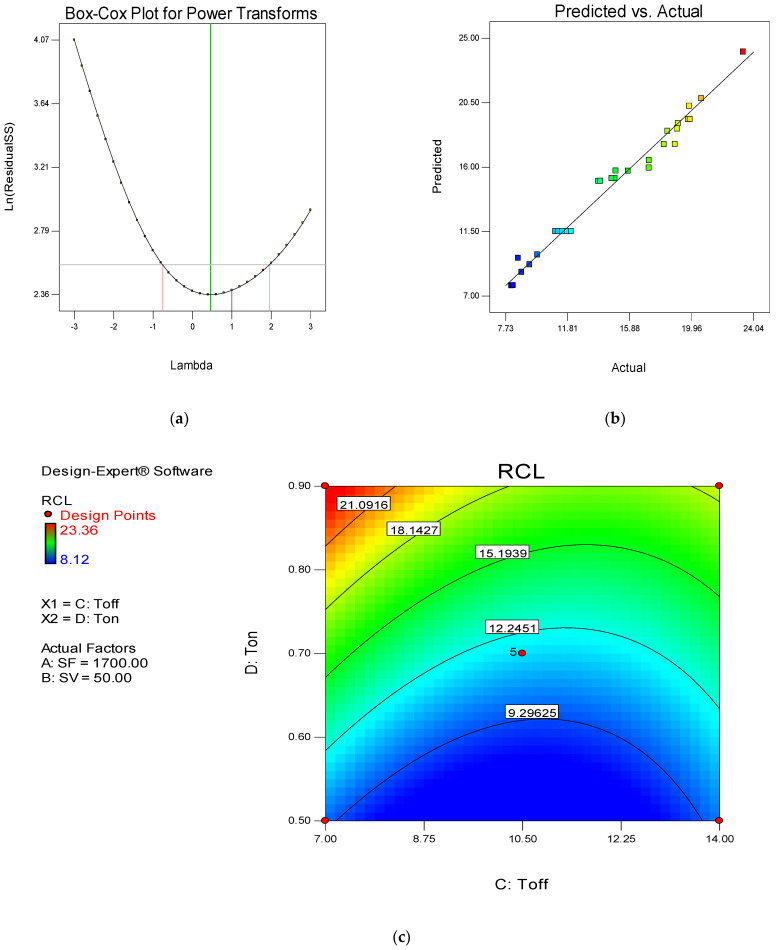
(**a**) Box–Cox transformation for RCL; (**b**) Predicted versus actual plot for RCL; (**c**) Contour plot for Toff and Ton in case of RCL.

**Figure 6:**
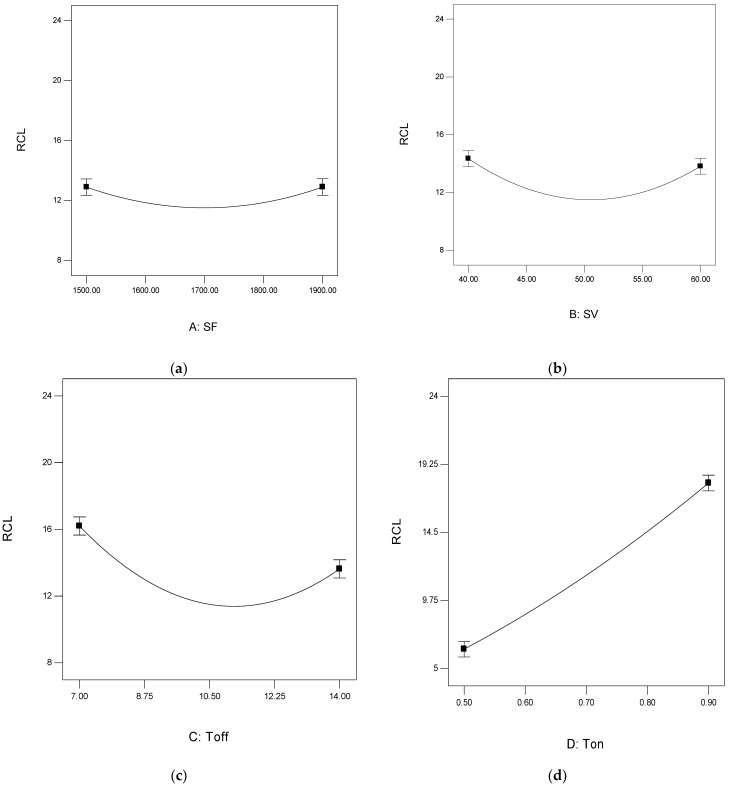
Graphs for RCL analysis. (**a**) Variation of RCL with SF; (**b**) Variation of RCL with SV; (**c**) Variation of RCL with Toff; (**d**) Variation of RCL with Ton.

The Box–Cox plot of power transformation shows that the recommended value of λ is 1 (Figure 5a). Thus, during the analysis of RCL, ‘1’ is used as the power of response data. Figure 5b represents the predicted versus actual plot for RCL. The clustering of values near the straight line exhibits a sign of good ANOVA, which is desired for the analysis. Figure 6c depicts the contour plot of RCL for the interaction of Toff and Ton. For RCL, the lower, the better type quality attribute is followed. The minimum RCL (8.12 µm) is obtained in the blue region of Figure 5c. Thus, high Toff and low Ton is desired for low RCL. However, with the increase on Ton value up to maximum (0.9 µs), keeping Toff (7 µs) at its lowest value, the maximum RCL of 23.36 µm is obtained (left top corner shows the red color with 23.36 µm value) [54]. The mathematical model developed after the analysis of RCL is given in Equation (8).

RCL = + 184.72565 − 0.11775 × SF − 2.60308 × SV − 4.81395 × T_off_ + 27.53042 × T_on_ − 2.00000 × T_off_ × T_on_ + 3.46375E – 005 × SF^2^ + 0.025767 × SV^2^ + 0.27841 × T_off_^2^ + 16.04375 × T_on_^2^ (8)

Figure 6a,b shows the variation of RCL concerning the SF and SV. It was observed from the figures that a negligible variation was found with these two parameters. The P-value of these two parameters is greater than 0.05, which reveals its non-significance. The research work done by Manjaiah et al. [57] shows a similar pattern while machining the D2 tool steel on WEDM. Due to low discharge energy, an increase in Toff value decreases the RCL (From 16.2 to 13.6 μm). Therefore, less material is melted and solidified at low discharge energy, which is the main reason for the recast layer. The results obtained in the present research work are very similar to the machining of NiTi smart alloy by Shandilya et al. [58]. Therefore, less material is deposited on the machined surface at low discharge energy, and small RCL is observed. Figure 6d depicts the variation of RCL with Ton, and it was found that with the increase in Ton value, the discharge energy in the circuit increases. This high discharge energy increases the molten material. Consequently, the RCL value increases from 6.34 to 17.94 μm with the re-solidified material [58].

### 4.3. Multiple Performance Measure Optimization by MOPSO

**Figure 7:**
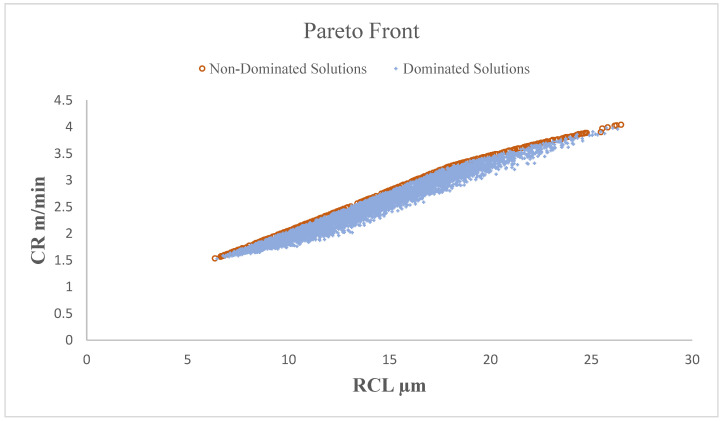
Pareto optimal front suggested by MOPSO.

In the present investigation, machining variables for WEDM are optimized for the machining of AZ31 alloy. The multiple objectives considered in the present study include the cutting rate (CR) and recast layer thickness (RCL). The second-order regression equations for both the objectives obtained from the response surface methodology are the objective function equations in the MOPSO algorithm. The bounds on the process variables are implemented as per Table 3. The Pareto optimal front is shown in Figure 7. Numerous trials were performed to finalize the MOPSO control factors. The selected values of the factors include the following:

- Swarm size = 100;
- Number of iterations = 150;
- Size of external repository = 200;
- Acceleration coefficients C1 = 1 and C2 = 1; and
- The minimum and maximum values of inertia are 0.1 and 0.4, respectively.

The experiments are performed on an Intel(R) Pentium(R) machine with processor I-7, 2.6 GHz, and the execution time is less than one minute.

### 4.4. Confirmation Experiments

The experiments (confirmation) are conducted on the predicted setting of the process variables suggested by MOPSO. From Table 7, a random set of experiments are selected for validation. Table 8 gives the setting of process variables for four different sets of experiments with the predicted solutions. The machine tool is set on these process variable values with nearly feasible values due to machine adjustments. For example, the suggested experimental set according to the first set is as follows:

SF: 183; SV: 42.163; Toff: 7.349; and Ton: 0.896.

However, due to machine tool limitation, experiments are performed on:

SF: 183; SV: 42 V; Toff: 7; and Ton: 0.9.

The experimental results provide a close agreement with the predicted solution. Therefore, the hybrid approach of RSM-MOPSO can be applied successfully to optimize the process variable of WEDM during the machining of AZ31 alloy.

## 5. Morphological Analysis of Machined Surface

**Figure 8:**
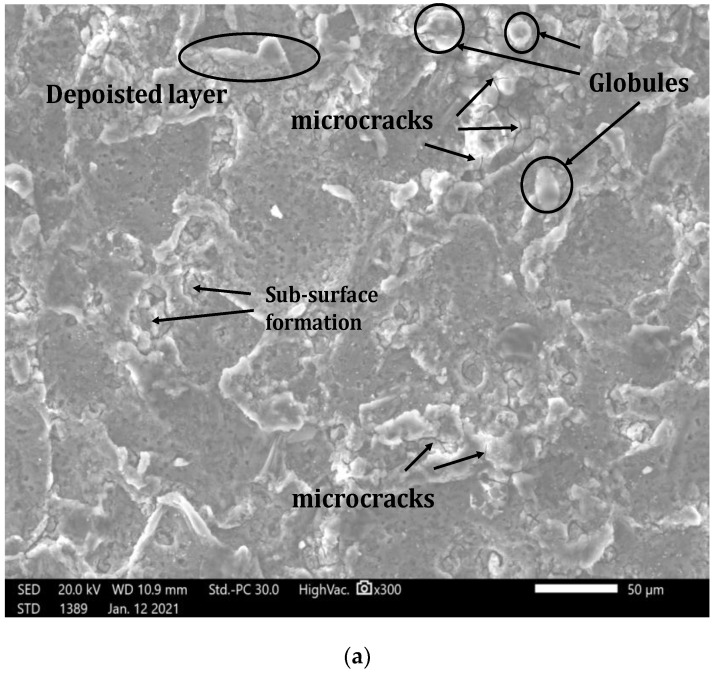

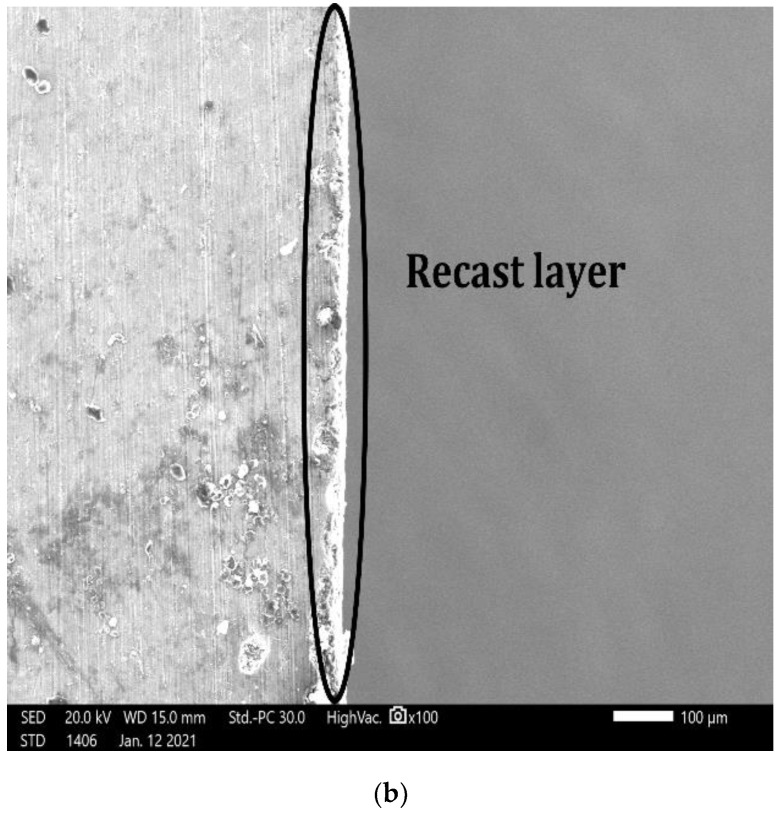
(**a**) Surface morphology. (**b**) Recast layer of AZ31 machined at SF: 1830; SV: 42 V; Toff: 7 µs; Ton: 0.9 µs.

The surface morphology of AZ31 alloy machined at WEDM (SF: 1830; SV: 42 V; Toff: 7 µs; Ton: 0.9 µs) is studied by Jeol make SEM. Figure 8a shows the micro-cracks, deposited lumps, and debris. During machining at WEDM, the temperature increases to 10,000 °C, which is adequate to melt and vaporize the conductive material. However, due to the whole dielectric material not being flushed away, the remaining metal is deposited on the surface (machined) in the form of deposited lumps. Some of the air bubbles also become impacted and become the reason for debris [59]. The recast layer (Figure 8b) is induced due to molten metal’s rapid heating and cooling. The reason for the surface cracks and large-size debris is the high value of applied erosive power [60].

## 6. Conclusions

The AZ31alloy was machined successfully by wire-cut electric discharge machining. The cutting rate and recast layer thickness were investigated while designing the servo feed, pulse on-time, servo voltage, and pulse off-time with the BBD of RSM. The ANOVA was performed to determine the influence of machining variables and the empirical models developed for CR and RCL using MOPSO. The following finding can be drawn from the present research:

- The proposed hybrid approach of optimization is a reliable method to predict the WEDM responses. ANOVA results show that the Ton is the most significant process variable for CR and RCL. Furthermore, an improvement in the response characteristics value is obtained after the confirmation test.
- The hybrid approach suggested different solutions for various response characteristics selection. Based on that, SF: 1830; SV: 42 V; Toff: 7 µs; Ton: 0.9 µs are suggested for maximum CR (3.18 m/min), and SF: 1720; SV: 51 V; Toff:10.5 µs; Ton: 0.5 µs are suggested for minimum RCL (6.37 µm).
- The proposed approach can help the decision-maker select process variables depending upon their requirement of response characteristics during the machining of AZ31 alloy.
- The SEM micrograph shows the debris, deposited lumps, and micro-cracks at the optimized process variables.

The accuracy of the results obtained from the hybrid approach of RSM-MOPSO is restricted due to the limited range of process parameters. However, more experiments can eliminate this limitation after widening the scope of process variables and selecting more machining variables and machining environments. In addition, this hybrid approach can optimize other responses, namely surface quality, residual stresses, microhardness, a circularity of intricate profiles, etc. Therefore, this approach can be used for traditional and other non-traditional manufacturing processes.

## Figures and Tables

**Table 1:** Properties of work material.

Characteristics	Value
Density (g/cm^3^)	1.77
Thermal conductivity (W/mK)	96
Elastic modulus (GPa)	45
Co-efficient of thermal expansion (µm/m°C)	26
Tensile strength (MPa)	260
Poisson’s ratio	0.35
Hardness (HRB)	49

**Table 2:** AZ31 alloy, chemical composition.

Element	Al	Zn	Mn	Cu	Si	Fe	Ca	Ni	Mg
Percentage	3.4	1.2	0.20	0.05	0.1	0.005	0.04	0.005	Balance

**Table 3:** Range and levels of process variables.

Sr. No.	Process Variable (Notation)	Units	Range of Parameters		Levels		
			Lower Limit	Upper Limit	1	2	3
1	Servo feed (SF)	-	1500	1900	1500	1700	1900
2	Servo voltage (SV)	V	40	60	40	50	60
3	Pulse off-time (Toff)	µs	7	14	10.5	7	14
4	Pulse on-time (Ton)	µs	0.5	0.9	0.5	0.7	0.9

**Table 4:** Experimental design and corresponding results.

Std Order	Run Order	SF	SV	Toff	Ton	RCL (µm)	CR (m/min)
1	24	1500	40	10.5	0.7	15.79	1.56
2	5	1900	40	10.5	0.7	14.98	1.61
3	26	1500	60	10.5	0.7	14.92	1.5
4	17	1900	60	10.5	0.7	14.71	1.53
5	19	1700	50	7	0.5	8.56	0.73
6	11	1700	50	14	0.5	9.82	0.63
7	29	1700	50	7	0.9	23.36	2.81
8	23	1700	50	14	0.9	19.02	2.17
9	28	1500	50	10.5	0.5	8.12	0.69
10	16	1900	50	10.5	0.5	8.23	0.71
11	15	1500	50	10.5	0.9	19.75	2.32
12	6	1900	50	10.5	0.9	19.86	2.4
13	22	1700	40	7	0.7	19.08	1.94
14	2	1700	60	7	0.7	18.39	1.83
15	27	1700	40	14	0.7	17.17	1.76
16	25	1700	60	14	0.7	17.16	1.7
17	7	1500	50	7	0.7	18.15	1.88
18	8	1900	50	7	0.7	18.87	1.91
19	20	1500	50	14	0.7	13.84	1.3
20	21	1900	50	14	0.7	13.97	1.43
21	14	1700	40	10.5	0.5	9.31	0.82
22	1	1700	60	10.5	0.5	8.79	0.74
23	3	1700	40	10.5	0.9	20.61	2.53
24	13	1700	60	10.5	0.9	19.81	2.43
25	9	1700	50	10.5	0.7	11.01	1.3
26	18	1700	50	10.5	0.7	11.22	1.34
27	12	1700	50	10.5	0.7	11.77	1.29
28	10	1700	50	10.5	0.7	11.48	1.21
29	4	1700	50	10.5	0.7	12.04	1.24

**Table 5:** Pooled ANOVA for CR.

Source	SS *	Df *	MS *	F-Value	*p*-Value *	
Model	10.04	9	1.12	150.82	<0.0001	significant
SF	9.63 × 10^−3^	1	9.63 × 10^−3^	1.3	0.2678	
SV	0.02	1	0.02	2.71	0.1164	
Toff	0.37	1	0.37	50.18	<0.0001	
Ton	8.91	1	8.91	1205.12	<0.0001	
Toff × Ton	0.073	1	0.073	9.86	0.0054	
SF^2^	0.058	1	0.058	7.9	0.0111	
SV^2^	0.35	1	0.35	47.9	<0.0001	
Toff^2^	0.41	1	0.41	55.35	<0.0001	
Ton^2^	0.082	1	0.082	11.09	0.0035	
Residual	0.14	19	7.39 × 10^−3^			
Lack of Fit	0.13	15	8.66 × 10^−3^	3.29	0.129	not significant
Pure Error	0.011	4	2.63 × 10^−3^			
Cor Total	10.18	28				

*p*-value—probability value; df—degree of freedom; MS—mean square; * SS—a sum of square.

**Table 6:** Pooled ANOVA for RCL.

Source	SS	df	MS	F-Value	*p*-Value	
Model	534.76	9	59.42	103.76	<0.0001	significant
SF	2.08 × 10^−4^	1	2.08 × 10^−4^	3.64 × 10^−4^	0.985	
SV	0.83	1	0.83	1.45	0.2428	
Toff	19.84	1	19.84	34.65	<0.0001	
Ton	403.45	1	403.45	704.5	<0.0001	
Toff × Ton	7.84	1	7.84	13.69	0.0015	
SF^2^	12.45	1	12.45	21.74	0.0002	
SV^2^	43.07	1	43.07	75.21	<0.0001	
Toff^2^	75.45	1	75.45	131.75	<0.0001	
Ton^2^	2.67	1	2.67	4.66	0.0438	
Residual	10.88	19	0.57			
Lack of Fit	10.2	15	0.68	3.98	0.0956	not significant
Pure Error	0.68	4	0.17			
Cor Total	545.65	28				

**Table 7:** Predicted solutions suggested by MOPSO.

Sr. No.	SF	SV	Toff	Ton	CR (m/min)	RCL (µm)
1	1833.07	42.163	7.349	0.896	3.950	25.389
2	1675.35	57.909	7	0.896	3.905	25.329
3	1687.75	57.298	7.015	0.9	3.901	25.175
4	1869.28	58.411	7.562	0.897	3.896	25.092
5	1669.8	56.698	7	0.9	3.887	25.044
6	1709.22	56.313	7	0.9	3.880	24.897
7	1680.79	57.691	7.215	0.9	3.867	24.794
8	1825.64	42.057	7.436	0.886	3.866	24.710
9	1712.67	55.944	7.067	0.9	3.857	24.612
10	1695.54	57.587	7.246	0.897	3.843	24.556
11	1720.82	51.415	10.497	0.5	1.529	6.370
12	1741.25	51.724	10.491	0.503	1.546	6.502
13	1716.89	51.625	10.875	0.510	1.561	6.664
14	1699.84	52.306	10.692	0.512	1.568	6.714
15	1728.42	52.182	9.860	0.510	1.584	6.768
16	1725.1	50.518	10.791	0.522	1.601	6.897
17	1725.51	50.968	10.259	0.525	1.619	6.980
18	1742.24	50.201	10.034	0.525	1.631	7.056
19	1729.07	49.213	10.739	0.533	1.642	7.178
20	1734.12	48.986	10.729	0.537	1.662	7.318
21	1696.5	48.736	10.457	0.815	2.820	15.148
22	1701.08	49.841	10.813	0.824	2.837	15.209
23	1728.06	51.389	10.704	0.823	2.841	15.252
24	1740.52	49.286	11.286	0.831	2.859	15.360
25	1704.67	49.741	11.024	0.833	2.869	15.408
26	1734.02	49.703	10.731	0.833	2.895	15.587
27	1750.68	53.258	9.2744	0.9	3.414	19.573
28	1716.78	51.684	9.0931	0.9	3.418	19.606
29	1741.18	49.572	10.446	0.681	2.210	11.054
30	1736.91	50.399	10.530	0.686	2.223	11.137

**Table 8:** Confirmation experiments performed at random settings suggested by MOPSO.

Sr. No.	SF	SV	Toff	Ton	Predicted Solution		Experimental Results	
					CR (m/min)	RCL (µm)	CR (m/min)	RCL (µm)
1	1833.07	42.163	7.349	0.896	3.950	25.389	3.92	24.67
11	1720.82	51.415	10.497	0.5	1.529	6.370	1.61	6.45
18	1742.24	50.201	10.034	0.525	1.631	7.056	1.68	7.18
23	1728.06	51.389	10.704	0.823	2.841	15.252	2.92	15.11

## Data Availability

The data presented in this study are available on request from the corresponding author.

## Nomenclature

Analysis of variance—ANOVA	Pulse on-time—Ton
Box–Behnken design—BBD	Recast layer—RCL
Cutting rate—CR	Response surface methodology—RSM
Multi-Objective Optimization—MOO	Servo feed—SF
Multi-Objective Particle Swarm Optimization—MOPSO	Servo voltage—SV
ϵ0, ϵk, ϵkk, and ϵkn—Regression coefficients	m—Number of process variables
A—Process variables	k—Level of process variables
Gbest—Global Best	Pbest—Particle best
x—Position of particle	v—Velocity of particle
c1 and c2—Coefficients	w—Inertia weight
Pulse off-time—Toff	Wire-cut Electric Discharge Machining—WEDM
